# Changes in Vaginal Microbiome Diversity in Women With Polycystic Ovary Syndrome

**DOI:** 10.3389/fcimb.2021.755741

**Published:** 2021-11-03

**Authors:** Chaoyi Lu, Hui Wang, Jihong Yang, Xinyue Zhang, Yao Chen, Ruizhi Feng, Yun Qian

**Affiliations:** ^1^ Reproductive Medical Center of Second Affiliated Hospital of Nanjing Medical University, Nanjing, China; ^2^ Department of Histology and Embryology, School of Basic Medical Sciences, Nanjing Medical University, Nanjing, China; ^3^ State Key Laboratory of Reproductive Medicine, Second Affiliated Hospital of Nanjing Medical University, Nanjing Medical University, Nanjing, China

**Keywords:** polycystic ovary syndrome, vaginal, microbiome, 16s rRNA gene sequencing, next-generation sequencing

## Abstract

Polycystic ovary syndrome (PCOS) is a complex endocrine disorder that affects women. It can be accompanied by many clinical manifestations that can vary between individuals. Previous studies have found that there are specific changes in the intestinal flora of PCOS patients, and interventions to modify the intestinal flora can significantly improve the symptoms of PCOS. Women with PCOS have a higher incidence of vaginitis compared to healthy women. Few studies to-date have focused on investigating vaginal flora. Here, we aimed to explore distribution changes of the vaginal microbiome in PCOS patients. We recruited 42 PCOS patients (T-PCOS) and 24 healthy controls (T-control). 16s rRNA gene sequencing was used to sequence their vaginal microbiome. Normally, Lactobacillus was dominated in vaginal. *Lactobacillus-*dominated-type vaginal microbiome in T-PCOS and T-control (L-PCOS and L-control) and non-*Lactobacillus-*dominated-type vaginal microbiome in T-PCOS and T-control (N-PCOS and N-control) were analyzed separately. A total of 655 operational taxonomic units were detected in this sequencing, including 306 unique to T-PCOS, 202 unique to T-control, and 147 common between the two groups. At the genus level, *Lactobacillus* accounted for more than 70% of the total microbiome. Observed species (P = 0.021), Chao1 index (P = 0.020), and ACE index (P = 0.023) decreased significantly in L-PCOS. Principal component analysis showed no statistically significant differences among the subgroups. There were significant statistical differences in principal coordinate analysis in the Jaccard distance between the T-PCOS and T-control groups and between the L-PCOS and L-control groups. Linear discriminant analysis effect size found that *Enterococcus* and *Actinomycetes* were significantly different in the T-PCOS group. *Atopobium* and *Actinomyces* were statistically significantly different in patients with L-PCOS and N-PCOS group, respectively. Environmental factor analysis found that *Ezakiella* was significantly negatively correlated with age, while *Streptococcus* was significantly negatively correlated with follicle stimulating hormone. There were statistically significant differences between PCOS patients and healthy women in the vaginal microbiome, regardless of the abundance of *Lactobacillus*. Alpha diversity of vaginal microbiome decreased markedly in PCOS patients when it was dominated by *Lactobacillus* spp. *Actinomyces* could be a potential biomarker to identify PCOS. *Streptococcus* may have an impact on the pathological changes in PCOS by affecting the female reproductive endocrine environment.

## Introduction

Polycystic ovary syndrome (PCOS) is a complex endocrine disorder that affects many women. It can be accompanied by many other clinical manifestations, including menstrual disorders, hyperandrogenemia, and multiple ovarian cysts (polycystic ovary); these manifestations can vary between individuals. Furthermore, women with PCOS have a higher incidence of insulin resistance, metabolic syndrome, periodontitis, and vaginitis compared to healthy women (Azziz, 2018). The pathogenesis of PCOS still is unknown. Recently, many studies have found specific changes in intestinal flora in PCOS patients, and interventions to normalize abnormal intestinal flora can significantly improve the symptoms of PCOS (Torres et al., 2019; He and Li, 2020). However, little research to date has focused on associations between vaginal flora and PCOS.

In recent decades, next-generation sequencing (NGS) technologies, such as 16s rRNA gene sequencing, has been widely used in intestinal microbiome studies (Song et al., 2018). In 2017, Qiao et al. (Chen et al., 2017) have conducted a multi-center prospective study to investigate the microbial distribution of the female reproductive system in Chinese women. They demonstrated the presence of microorganisms in the endometrium and fallopian tubes. Subsequently, 16s rRNA gene sequencing was used to sequence the microbiome of the lower genital tract (Tu et al., 2020) and endometrium (D’Ippolito et al., 2018), confirming the application of these technologies in studying the female reproductive system.

Vaginal secretion sampling has advantages of being fast and noninvasive. The vaginal microbiome is a unique flora and in a healthy individual, it is mainly dominated by *Lactobacillus* spp. Other bacteria account for less than 10% of the overall microbial flora, and they are present in very low abundance (Greenbaum et al., 2019), rendering it difficult to investigate the distribution of microorganisms in the vaginal microbiome using the traditional vaginal secretion method. NGS technologies may solve this problem and greatly improve the feasibility of related research.

This study aimed to explore distribution changes of the vaginal microbiome in PCOS patients in order to provide direction and theoretical foundation for follow-up treatment. Meanwhile, we analyzed differences in *Lactobacillus-*dominated and non-*Lactobacillus*-dominated patients.

## Materials and Methods

### Study Population

Forty two newly diagnosed PCOS patients were included in this study. All patients were treated in the reproductive medicine center of the Second Affiliated Hospital of Nanjing Medical University, China, between January 2018 and December 2020. This study was reviewed and approved by the Reproductive Medicine Ethics Committee of the Second Affiliated Hospital of Nanjing Medical University. The control group consisted of 24 healthy, age-matched volunteers. In this study, the Rotterdam criteria were adopted for the diagnosis of PCOS—1. Abnormal ovulation (oligomenorrhea or amenorrhea); 2. Hyperandrogenism (clinical or biological), and 3. Polycystic ovary morphology. Meanwhile, diseases that cause abnormal ovulation, amenorrhea, and hyperandrogenemia were excluded, such as thyroid dysfunction, Cushing’s syndrome, ovarian androgen-secreting tumors, and hyperprolactinemia (Goodman et al., 2015a; Goodman et al., 2015b). The exclusion criteria were as follows: 1. malformation or organic disease of the reproductive system; 2. cancer or operative history, radiotherapy, and chemotherapy experience; 3. sexual behavior or vaginal douche within 5 days before sample collection; 4. smoking and/or drinking; and 5. consumption of antibiotics, antidiabetics, or oral contraceptives within one month before enrolment. Informed consent was signed by all participants.

### Vaginal Secretion Sampling

Swabs were collected within 3–7 days after menstruation (during the first visit for patients with amenorrhea). Firstly, the vulva was wiped twice with normal saline. Subsequently, the cervix was fully exposed with a disposable sterile vaginal speculum. Using a sterile vaginal swab, secretions of the vaginal posterior fornix were swabbed, and attention was paid to avoid urine and blood contamination. The samples were treated with liquid nitrogen within 30 min after sampling and then stored at -80°C for future studies.

### 16s rRNA Gene Sequencing

16s rRNA Gene Sequencing was performed by Shanghai Genesky Biotechnologies Inc.

Bacterial DNA was extracted from vaginal secretions using the FastDNA™ spin kit for soil (MP Biomedicals, USA) (Li et al., 2018).

The 16s rRNA hypervariable region V4 in bacterial DNA samples were amplified, using the forward primer: 515-F:5’-GTGCCAGCMGCCGCGGTAA-3’, and reverse primer: 806-R:5’-GGACTACHVGGGTWTCTAAT-3’. A 50 μL reaction volume was prepared, consisting of 5 μL 10 × polymerase chain reaction (PCR) buffer, 4 μ L of 2.5 mM dNTP mix, 2 μL of forward primer, 2 μL of reverse primer, 1 μL of template DNA,1 μL of Taq enzyme, and ddH_2_O up to 50 μL. A PCR reaction for each DNA sample was performed in triplicate. The three PCR products obtained from each sample were combined and purified using the AMPure XP for PCR Purification Kit (Beckman Kurt Co. Ltd., USA). Purified PCR products were stored at –20°C.

Specific tag sequences were introduced into the library using high-fidelity PCR. Multiple samples can be mixed during online sequencing, and subsequent bioinformatics processing can distinguish samples with different tag sequences. The amplified DNA product was detected by agarose gel electrophoresis and purified using the AMPure XP for the PCR Purification Kit (Beckman Kurt Co. Ltd., USA) to build the original library. The concentration of the library was diluted according to quantitative results of agarose gel electrophoresis. The library was then quantified using the Invitrogen Qubit3.0 Spectrophotometer (Thermo Fisher Scientific, USA). Samples were blended in a corresponding ratio (mole ratio) according to sequencing flux requirements of different samples. The length of the inserted fragment of the sequencing library was measured using the Agilent 2100 Bioanalyzer (Agilent Technologies, USA). No non-specific amplification was observed between 120–200 bp. The concentration of the sequencing library was quantified again. Finally, the library was sequenced using the Illumina NovaSeq 6000 platform (Illumina, USA) and the double-ended sequencing strategy of SP XP (PE250).

### Statistical Analysis

QIIME2 (https://qiime2.org/) was used to remove possible adapter sequences and primers, filter the data quality, and annotate the operational taxonomic units (OTUs) (confidence: 0.8). SPSS (version 22.0; SPSS Inc., USA), and Stata (https://www.stata.com/) were used for statistical analysis. R (version 4.1.1) was used to map graphs. Independent sample t-test or k-independent sample nonparametric tests were used to determine differences between groups. The chi-square test was used for the hypothesis test, and the nonparametric Kruskal-Wallis rank sum test was used for multi-sample analysis. Interference factors were adjusted using a zero-inflated Poisson regression. The Pearson correlation coefficient was used to determine a correlation between microbiome and environmental factors, and a heat map was generated using Multiple Experiment Viewer. The P-value was adjusted using the Benjamini-Hochberg false discovery rate, and the bilateral P value (< 0.05) was statistically significant.

## Results

### Clinical Epidemiological Information

Sixty eight volunteers were recruited between January 2018 to December 2020, including 43 volunteers who met the diagnosis of PCOS and 25 healthy volunteers who received assisted reproductive technology due to male infertility. One patient in the PCOS group was excluded because the quality of their sample was poor, and one individual in the control group did not meet the criteria as a healthy control. Therefore, 66 patients were included in the study: 42 in the total PCOS group (T-PCOS) and 24 in the total control group (T-control). There were no significant differences in age, height, and estradiol levels between the two groups. The weight, luteinizing hormone (LH), testosterone (T), body mass index (BMI), antral follicle count (AFC), and LH/FSH ratio significantly increased in the PCOS group, while there was a significant decrease in levels of follicle stimulating hormone (FSH) (**Table 1**).

**Table 1:** Clinical epidemiological information of all the volunteers.

	PCOS	control	P
age	28.071±3.872	29.458±4.549	0.194
height	1.612±0.048	1.618±0.06	0.687
weight	66.833±10.987	57.017±6.071	**<0.001**
FSH	6.125±1.282	7.487±1.335	**<0.001**
LH	7.615±5.815	5.184±2.313	**0.02**
E_2_	61.667±38.085	53.583±16.955	0.329
T	0.651±0.243	0.496±0.155	**0.007**
BMI	25.758±4.425	21.790±2.273	**<0.001**
AFC	28.619±7.660	16.458±6.171	**<0.001**
LH/FSH	1.225±0.810	0.710±0.340	**0.001**

FSH, Follicle-stimulating hormone; LH, luteinizing hormone; E2, estradiol; T, testosterone; BMI, Body mass index; AFC, antral follicle count. Data are presented as mean values and standard error.

The value should be bold if it was less than 0.05.

### OTUs Analysis

**Figure 1:**
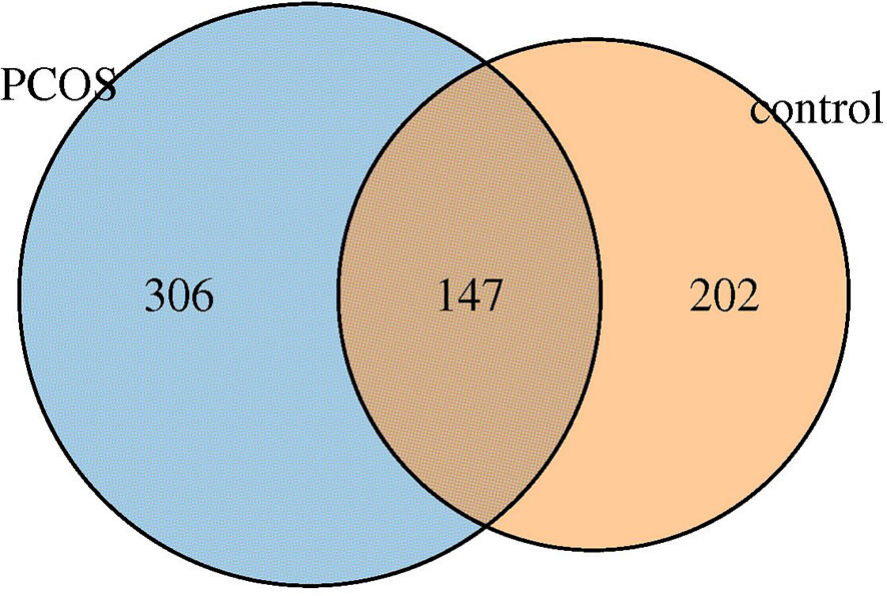
Venn diagram of total polycystic ovary syndrome (PCOS) group and total control group.

**Figure 2:**
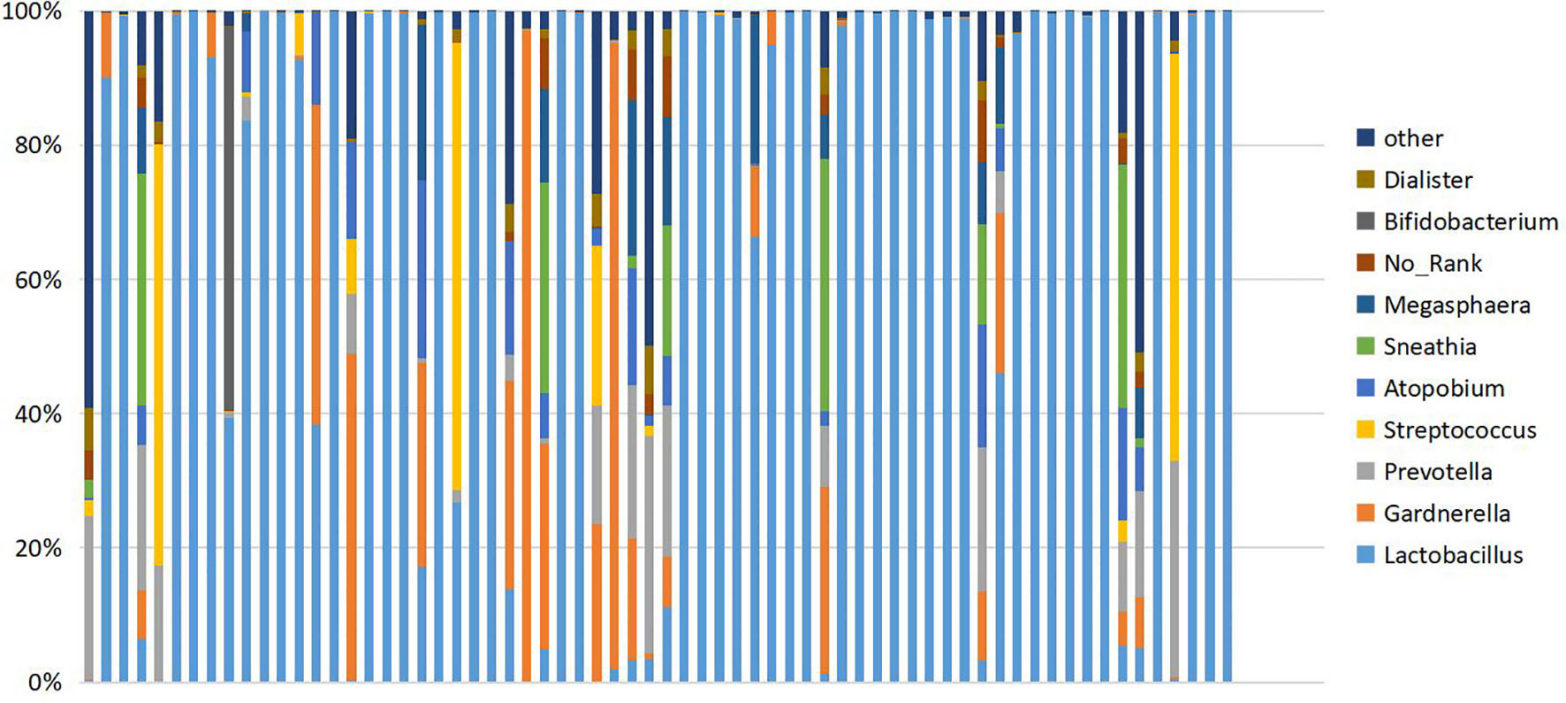
Cumulative histogram showing the relative abundance of the vaginal microbiome at genus level. Ten species showing the highest relative abundance are displayed.

Six hundred fifty five OTUs were detected in this sequencing, including 306 unique to T-PCOS, 202 unique to T-control, and 147 common between the two groups (**Figure 1**). Results showed that at the genus level, *Lactobacillus* accounted for more than 70% of the total microbiome. In the test population, 43 cases were dominated by *Lactobacillus* (relative abundance ≥ 70%), including 25 patients of the T-PCOS group and 18 patients of the T-control group; 23 patients had an abundance of non-*Lactobacillu*s bacteria (*Lactobacillus* relative abundance < 70%), 17 of these were from the T-PCOS group, and 6 from the T-control group (**Figure 2**). The chi-square test showed no significant statistical difference in the relative abundance of *Lactobacillus* between the T-PCOS and T-control groups.

The vaginal microbiome can be divided into *Lactobacillus* dominant and non-*Lactobacillus* dominant types, depending on different proportions of *Lactobacillus* in samples. The composition of the two microbial floras differs greatly, which may have an impact on diversity analysis. Therefore, in the *Lactobacillus* dominant type group, there were 25 cases in T-PCOS, now referred to as the L-PCOS group, and 18 cases in T-control, now referred to as the L-control group. From the non-*Lactobacillus* dominant type group, there were 17 cases in the T-PCOS group and 6 cases in the T-control group, now referred to as the N-control group.

### Alpha Diversity Analysis

**Figure 3:**
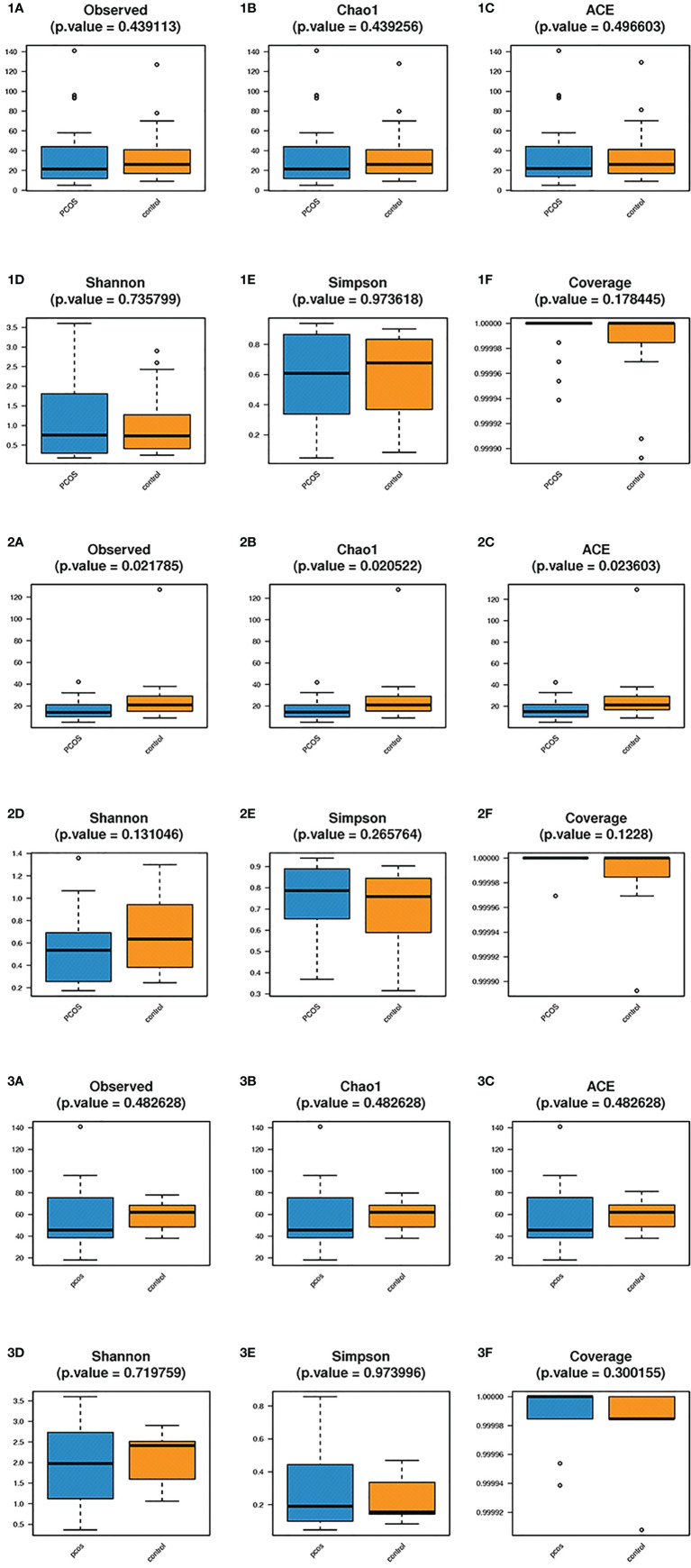
Alpha diversity analyzes between the total polycystic ovary syndrome (PCOS) and total control groups, L-PCOS and L-control groups, N-PCOS and N-control groups. Alpha diversity analyzes between the total PCOS and total control groups **(1A–F)**; between the L-PCOS and L-control groups: **(2A–F)**; and between the N-PCOS and N-control groups **(3A–F)**.

Alpha diversity analysis was used to estimate the abundance and diversity of species within samples. Observed species, Chao1 index, and ACE index were used to estimate the richness of the microbial flora in the samples. The observed species (P = 0.021), Chao1 index (P = 0.020), and ACE index (P = 0.023) of the L-PCOS group reduced significantly compared to the L-control. The microbiome richness of the L-control group was remarkably higher than that of the L-PCOS group [**Figures 3 (2A–C)**]. No statistically significant difference was observed between the T-PCOS and T-control groups [**Figures 3 (1A–C)**] or between the N-PCOS and N-control groups [**Figures 3 (3A–C)**]. The Shannon index and Simpson index were used to estimate the diversity of microbial flora in the samples, with the good’s cover age estimating sequencing depth, and there was no difference between the three subgroups [**Figures 3 (1D–F, 2D–F, 3D–F)**].

### Beta Diversity Analysis

Beta diversity analysis mainly includes principal component analysis (PCA) and principal coordinate analysis (PCoA). Adonis analysis was adopted to determine differences between groups, while linear discriminant analysis (LDA) effect size (LEfSe) analysis can be used to find biomarkers.

**Figure 4:**
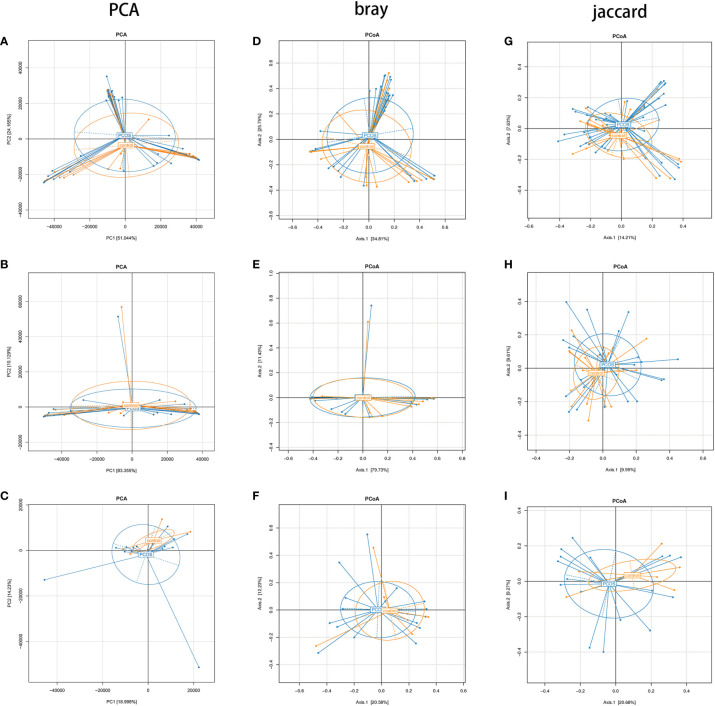
Principal component analysis and principal coordinate analysis (PCoA) between the total polycystic ovary syndrome (PCOS) and total control groups, L-PCOS and L-control groups, and N-PCOS and N-control groups. **(A–C)** PCA between total PCOS group and total control group, L-PCOS group and L-control group, N-PCOS group and N-control group; **(D–F)** PCoA in bray distance between total PCOS group and total control group, L-PCOS group and L-control group, N-PCOS group and N-control group; **(G–I)** PCoA in Jaccard distance between total PCOS group and total control group, L-PCOS group and L-control group, N-PCOS group and N-control group.

PCA showed no statistically significant differences in all subgroups (**Figures 4A–C**). In PCoA analysis, there was no statistically significant difference in bray distance between the T-PCOS and T-control groups (P = 0.587), but the said difference was noted in Jaccard distance (P = 0.014). However, there was an intergroup difference (R2 = 0.025) (**Figures 4D, G**). There was no statistically significant difference in bray distance between the L-PCOS and L-control groups (P = 0.843), while a statistically significant difference was observed in Jaccard distance (P = 0.003). This was also an intergroup difference (R2 = 0.041) (**Figures 4E, H**). No statistically significant differences in bray distance (P = 0.798) or Jaccard distance (P = 0.363) between the N-PCOS and N-control groups (**Figures 4F, I**) were seen.

**Figure 5:**
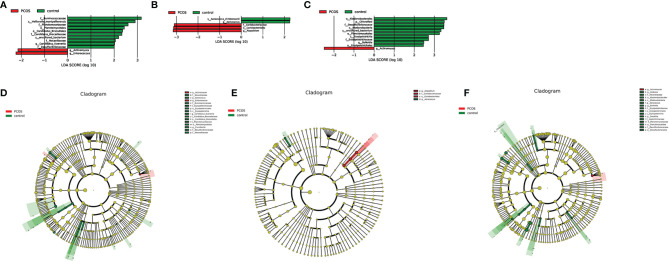
Linear discriminant analysis (LDA) score histogram and cladogram of the total polycystic ovary syndrome (PCOS) and total control groups, the L-PCOS and L-control groups, and the N-PCOS and N-control groups. **(A)** LDA score histogram of the total PCOS and total control groups; **(B)** LDA score histogram of the L-PCOS and L-control groups; **(C)** LDA score histogram of the N-PCOS and N-control groups; **(D)** LDA score cladogram of the total PCOS and total control groups; **(E)** LDA score cladogram of the L-PCOS and L-control groups; **(F)** LDA score cladogram of the N-PCOS and N-control groups.

As mentioned earlier, PCoA analysis was used to established beta diversity differences between groups. Similarly, LEfSe analysis was used to identify species with significant differences in PCOS groups as biomarkers for PCOS diagnosis. At the genus level, *Enterococcus* (LDA=2.14, P=0.013) and *Actinomycetes* (LDA=2.223, P=0.036) were statistically significantly different in the T-PCOS group while *Atopobium* (LDA=3.151, p=0.032) was statistically significantly different in the L-PCOS patients. *Actinomyces* (LDA=2.486, P=0.045) was statistically remarkably different in the N-PCOS group (**Figure 5** and **Table 2**).

**Table 2:** Linear discriminant analysis (LDA) scores and P values between_ the total polycystic ovary syndrome (PCOS) and total control groups, the L-PCOS and L-control groups, and the N-PCOS and N-control groups.

Taxonomy	LDA	P value	Group
g_Actinomyces	2.140	0.013	T-PCOS
g_Enterococcus	2.223	0.036	T-PCOS
f_Ruminococcaceae	3.165	0.011	T-control
f_Nocardiaceae	2.054	0.017	T-control
o_Planctomycetales	2.435	0.020	T-control
g_Alistipes.s_uncultured_bacterium	2.191	0.020	T-control
f_Planctomycetaceae	2.599	0.020	T-control
f_Desulfovibrionaceae	2.004	0.020	T-control
f_Candidatus_Brocadiaceae	2.335	0.020	T-control
g_Candidatus_Kuenenia	2.014	0.020	T-control
o_Candidatus_Brocadiales	2.365	0.020	T-control
s_Veillonella_montpellierensis	2.902	0.020	T-control
o_Erysipelotrichales	2.173	0.037	T-control
f_Erysipelotrichaceae	2.047	0.037	T-control
c_Erysipelotrichia	2.099	0.037	T-control
s_Aerococcus_christensenii	3.063	0.037	T-control
g_Aerococcus	3.051	0.037	T-control
g_Curvibacter	2.144	0.039	T-control
f_Moraxellaceae	2.022	0.045	T-control
g_Atopobium	3.151	0.032	L-PCOS
f_Coriobacteriaceae	3.094	0.046	L-PCOS
o_Coriobacteriales	3.132	0.046	L-PCOS
s_Aerococcus_christensenii	2.252	0.022	L-control
g_Aerococcus	2.242	0.022	L-control
g_Actinomyces	2.486	0.045	N-PCOS
g_Alistipes.s_uncultured_bacterium	3.334	0.005	N-control
o_Erysipelotrichales	2.454	0.015	N-control
f_Erysipelotrichaceae	2.708	0.015	N-control
c_Erysipelotrichia	2.708	0.015	N-control
g_Bulleidia	2.477	0.022	N-control
o_Ktedonobacterales	3.624	0.024	N-control
c_Ktedonobacteria	3.474	0.024	N-control
f_Desulfovibrionaceae	3.481	0.024	N-control
p_Chloroflexi	3.495	0.024	N-control
o_Planctomycetales	3.256	0.024	N-control
o_Desulfovibrionales	3.363	0.024	N-control
f_Planctomycetaceae	3.480	0.024	N-control
f_Rikenellaceae	3.383	0.031	N-control
g_Alistipes	3.075	0.031	N-control
s_Gemella_asaccharolytica	4.144	0.044	N-control
g_Sneathia	4.818	0.049	N-control
f_Leptotrichiaceae	4.821	0.049	N-control
s_Aerococcus_christensenii	3.567	0.049	N-control
g_Aerococcus	3.559	0.049	N-control

The predictive value of these species for the diagnosis of PCOS was determined using the receiver operating characteristic (ROC) curve. In the T-PCOS group, the area under curve (AUC) of *Actinomyces* was 0.6324, with 30.95% sensitivity and 95.83% specificity. The AUC of *Enterococcus* was 0.5833, with 16.67% sensitivity and 100% specificity. After combining the two bacteria, the AUC resulted in 0.6468 with 33.33% sensitivity and 95.83% specificity. In the L-PCOS group, the AUC of *Atopobium* was 0.6756, with 55.56% sensitivity and 88.00% specificity, while the AUC of *Actinomyces* was 0.7353, with 64.71% sensitivity and 83.33% specificity in the N-PCOS group.

### Environmental Factors Analysis

Correlations between various species and environmental factors were analyzed using Pearson’s correlation coefficient, which can evaluate species that are specifically affected by environmental factors and the way they impacted in. Taking the absolute value of Pearson correlation coefficient r > 0.3 and P value < 0.05 as the significance screening threshold, we can screen species significantly related to various environmental factors and showed the correlation between genus level species and environmental factors in the form of heat maps.

**Figure 6:**
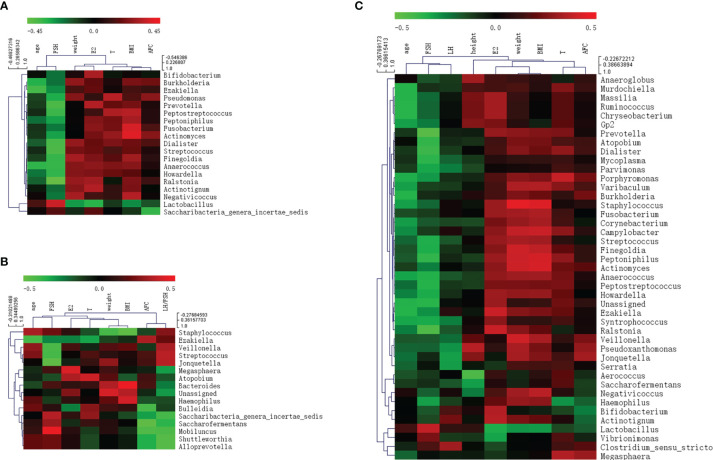
Heat map of Pearson correlation coefficient between vaginal microbiome and environmental factors. **(A)** Pearson correlation coefficient heat map between vaginal microbiome and environmental factors in total samples; **(B)** Pearson correlation coefficient heat map between vaginal microbiome and environmental factors in control samples; **(C)** Pearson correlation coefficient heat map between vaginal microbiome and environmental factors in polycystic ovary syndrome samples.

At the genus level, 19 species in total samples, 16 species in control samples, and 43 species in PCOS samples were significantly correlated with environmental factors. *Ezakiella* was significantly negatively correlated with age total group, P = 0.002; control group P = 0.044; PCOS, P = 0.022). *Streptococcus* was significantly negatively correlated with FSH (total group, P = 0.001; control group, P = 0.046; PCOS group, P = 0.027) in all groups (**Figure 6** and **Table 3**).

**Table 3:** Pearson correlation coefficient and P value between vaginal microbiome and environmental factors.

	Total (n=66)	Control (n=24)	PCOS (n=42)
***age***			
Anaerococcus	-0.334 (0.006)	-0.318	-0.320 (0.039)
Burkholderia	-0.314 (0.010)	-0.190	-0.353 (0.022)
Ezakiella	-0.370 (0.002)	-0.414 (0.044)	-0.352 (0.022)
Porphyromonas	-0.235	-0.099	-0.341 (0.027)
Unassigned	-0.216	-0.003	-0.350 (0.023)
Massilia	-0.289	–	-0.356 (0.021)
Murdochiella	-0.290	-0.182	-0.355 (0.021)
Ruminococcus	-0.289	–	-0.357 (0.020)
Chryseobacterium	-0.143	0.122	-0.357 (0.020)
***height***			
Aerococcus	0.013	-0.093	-0.432 (0.004)
Pseudoxanthomonas	-0.160	–	0.363 (0.018)
***weight***			
Prevotella	0.301 (0.014)	0.385	0.277
Peptoniphilus	0.303 (0.013)	0.101	0.399 (0.009)
Peptostreptococcus	0.313 (0.010)	0.306	0.246
Fusobacterium	0.330 (0.007)	0.136	0.320 (0.039)
Actinomyces	0.386 (0.001)	-0.136	0.405 (0.008)
Staphylococcus	0.254	-0.415 (0.044)	0.456 (0.002)
Finegoldia	0.297	0.109	0.402 (0.008)
Campylobacter	0.294	0.203	0.384 (0.012)
Veillonella	0.166	0.172	0.408 (0.007)
Varibaculum	0.284	0.242	0.346 (0.025)
***FSH***			
Lactobacillus	0.357 (0.003)	0.214	0.381 (0.013)
Prevotella	-0.386 (0.001)	-0.101	-0.464 (0.002)
Streptococcus	-0.392 (0.001)	-0.411 (0.046)	-0.341 (0.027)
Dialister	-0.383 (0.002)	-0.174	-0.392 (0.010)
Anaerococcus	-0.345 (0.005)	-0.233	-0.348 (0.024)
Finegoldia	-0.343 (0.005)	-0.198	-0.337 (0.029)
Peptostreptococcus	-0.354 (0.004)	-0.201	-0.353 (0.022)
Howardella	-0.337 (0.006)	-0.111	-0.332 (0.032)
Ralstonia	-0.443 (<0.001)	-0.384	-0.439 (0.004)
Pseudomonas	-0.304 (0.013)	-0.313	-0.164
Mobiluncus	0.103	0.472 (0.020)	-0.197
Jonquetella	-0.257	-0.480 (0.018)	-0.076
Atopobium	-0.243	-0.152	-0.378 (0.014)
Peptoniphilus	-0.286	0.011	-0.373 (0.015)
Mycoplasma	-0.182	0.043	-0.368 (0.017)
Unassigned	-0.198	-0.051	-0.359 (0.020)
***E2***			
Lactobacillus	-0.322 (0.008)	-0.360	-0.338 (0.028)
Prevotella	0.316 (0.010)	0.355	0.296
Bifidobacterium	0.303 (0.014)	–	0.352 (0.022)
Actinotignum	0.319 (0.009)	–	0.362 (0.019)
Ralstonia	0.289	0.062	0.396 (0.009)
Ezakiella	0.183	-0.257	0.395 (0.010)
***T***			
Peptostreptococcus	0.311 (0.011)	0.340	0.248
Pseudomonas	0.326 (0.008)	0.279	0.284
Megasphaera	0.188	0.027	0.354 (0.021)
Porphyromonas	0.218	0.065	0.388 (0.011)
***BMI***			
Peptostreptococcus	0.326 (0.008)	0.398	0.234
Fusobacterium	0.382 (0.002)	0.256	0.341 (0.027)
Actinomyces	0.399 (0.001)	-0.226	0.422 (0.005)
Negativicoccus	0.323 (0.008)		0.370 (0.016)
Staphylococcus	0.238	-0.472 (0.020)	0.436 (0.004)
Bacteroides	0.222	0.479 (0.018)	0.132
Peptoniphilus	0.296	-0.040	0.417 (0.006)
Finegoldia	0.283	0.096	0.374 (0.015)
Campylobacter	0.276	0.009	0.398 (0.009)
Varibaculum	0.286	0.287	0.349 (0.023)
***AFC***			
Saccharibacteria_genera_incertae_sedis	-0.343 (0.005)	-0.310	-0.265
Saccharofermentans	-0.196	-0.482 (0.017)	-0.123
***LH/FSH***			
Shuttleworthia	-0.278	-0.455 (0.025)	–
Alloprevotella	-0.264	-0.455 (0.025)	-0.250

FSH, Follicle-stimulating hormone; LH, luteinizing hormone; E2, estradiol; T, testosterone; BMI, Body mass index; AFC, antral follicle count. All Pearson correlation coefficients with P < 0.03 are listed, and the P value is displayed when it was less than P= 0.05.

## Discussion

In our study, the 16S rRNA gene sequencing technique was used to describe the characteristics of the vaginal microbiome in PCOS and healthy (control group) women. Alpha diversity, beta diversity, and environmental factor analyses were performed. As the vaginal microbiome was divided into *Lactobacillus* dominated and non-*Lactobacillus* dominated types, we further divided the PCOS group into the L-PCOS and N-PCOS groups, and the control group into the L-control and N-control groups.

In the alpha diversity analysis, the observed species, both the Chao1 and ACE indexes in the L-PCOS group decreased. The Shannon and Simpson indices showed a diminishing trend, but this was not statistically significant. Our results suggested that there were significant changes in the vaginal microbial environment in patients with PCOS dominated by *Lactobacillus*, which was reflected in the decline of the overall abundance of microbial flora and the declining trend of bacterial diversity. A previous study have found that the vaginal microbial diversity of PCOS patients was increased (Hong et al., 2020), but the study did not group patients based on dominance of *Lactobacillus*. This was the first report highlighting a significant decrease in microbiome abundance in PCOS patients without vaginitis, suggesting that the microbial population changed before the inflammation occurred. It is inferred that the change in the vaginal microbiome in PCOS patients may be related to the high incidence of vaginitis. This inference is supported by the results of previous studies on intestinal, oral, and vaginal flora (Liu et al., 2017; Guerra et al., 2018; Redelinghuys et al., 2020). The reduction in microbial abundance and diversity is significantly correlated with pathological changes, such as inflammation. High microbial abundance and diversity can make the environment more stable (Sze and Schloss, 2016). Chronic low-grade inflammation is an important risk factor involved in the occurrence and development of PCOS, which may promote hyperandrogenemia and insulin resistance and participate in pathological changes observed in PCOS (Shorakae et al., 2018). It is worth noting that previous studies have reported that inflammation and infection of the lower reproductive tract may affect function further up the reproductive tract (Rampersaud et al., 2012; Haahr et al., 2016; Ding et al., 2021). Patients with PCOS may have primary ovarian inflammatory injury, which affects the development and even occurrence of PCOS through the hypothalamic-pituitary-ovary axis. Because this was a cross-sectional study, the causal relationship between abnormal vaginal flora and PCOS could not be clarified. However, this is still a new perspective for studying the pathogenesis of PCOS.

In the beta diversity analysis, there were apparent differences in PCoA in the Jaccard distance between the total PCOS group and total control group, and in the L-PCOS and L-control groups, while no differences in the PCA and PCoA in bray distance were observed. Hong et al. (Hong et al., 2020) have discovered significant statistical differences in PCoA analysis of vaginal flora between PCOS and healthy people. However, there is also evidence suggesting that there is no statistically significant difference in beta diversity, for example, in intestinal flora and oral flora in PCOS patients (Lindheim et al., 2016; Rizk and Thackray, 2021). This contradiction may exist for several reasons. Firstly, the composition of microorganisms is often disturbed by influencing factors such as host age, BMI, and endocrine status. Meštrović T. et al. (2020) have observed that vaginal flora is affected by intestinal flora. Furthermore, the sample size was insufficient. According to Kelly et al. (2015), relatively robust statistical results can be obtained when the sample size of each group is 20 or above. Finally, it may be influenced by the different population included in different studies. PCOS is often accompanied by vaginitis, insulin resistance, and periodontitis. Some studies have excluded PCOS patients with complications, while others did not, or did not carry out further grouping analysis. These differences can make beta diversity analyses obtain different results. In this study, the proportion of PCOS patients with vaginitis was high (17/42). After secondary grouping, it was found that the difference in beta diversity mainly existed in the vaginal flora dominated by *Lactobacillus*. However, this result does not imply that there is no difference in the beta diversity of vaginal flora in PCOS patients under non-*Lactobacillus* dominated type conditions. The sample sizes of the N-PCOS and N-control groups were insufficient and need to be increased in future studies. In order to identify significantly different species of PCOS patients, we conducted LEfSe analysis, hoping to find biomarkers that could be used in the diagnosis of PCOS. *Actinomyces* and *Enterococcus* were enriched in T-PCOS. *Atopobium* was enriched in PCOS patients in the *Lactobacillus*-dominated type. *Actinomyces* was significantly increased in non-*Lactobacillus-*dominated PCOS patients. The ROC curve was used to evaluate the diagnostic significance of these bacteria for PCOS. The sensitivity and specificity of all of these species were low, although the LDA value was > 2 and P value < 0.05. The AUC of *Actinomyces* in the non-*Lactobacillu*s dominant type was > 0.7. However, the sample size needs expansion because of the small sample size of the N-PCOS and N-control groups. Moreover, *Actinomyces* has insufficient predictive ability in the T-PCOS and T-control groups, which suggests that *Actinomyces* may not be applicable to the general population.

Environmental factor analysis can explain the impact of environmental factors on vaginal flora. In patients with PCOS, there was an obvious correlation between the composition of the flora and FSH. The abundance of many anaerobes and facultative anaerobes was significantly negatively correlated with FSH, while *Lactobacillus* was significantly positively correlated with FSH levels. Serum concentrations of anti-Müllerian hormone, LH, and estrogen in PCOS patients were excessive, which would trigger negative feedback regulation to reduce the level of FSH. Low FSH levels are related to abnormal follicular development and non-ovulation in patients (Dewailly et al., 2016). This study revealed that the decrease in FSH was significantly correlated with an increase in the abundance of vaginal opportunistic pathogens, while the high abundance of *Lactobacillus* seemed to be conducive to maintaining the normal level of FSH. However, our study did not determine the ovulation rate. Further research is needed to track the ovulation rate, menstrual cycle, pregnancy rate, and other clinical indicators of PCOS patients to determine whether specific flora have an impact on the clinical manifestations of PCOS. In this study, LH only affected species in patients with PCOS. It is possible that LH levels in the control group include here were primarily normal, while very few LH levels in PCOS patients were extremely elevated, which may interfere with the statistical results.

In addition, *Streptococcus* was significantly negatively correlated with FSH levels in all three groups. Environmental factors may partly explain changes in the abundance of vaginal flora in patients with PCOS. *Streptococcus* is a common opportunistic pathogen of suppurative inflammation, which is prevalent in the feces and nasopharynx. Group B *Streptococcus*, a species of *Streptococcus*, is one of the main pathogens of perinatal infection in women (Baker, 2013). Previous studies on vaginal flora have showed that the abundance of *Streptococcus* is significantly positively correlated with age and estradiol (Xu et al., 2020), suggesting that it may be related to ovarian dysfunction. Our study found that *Streptococcus* was negatively correlated with FSH. This result supports the hypothesis that *Streptococcus* may influence sex hormones and ovarian function. Thus, the vaginal microbiome plays an important role in maintaining a healthy female reproductive system.

## Conclusion

Changes in the vaginal microbiome diversity are closely related to PCOS. There were significant differences in the vaginal microbiome between PCOS patients and healthy women, regardless of their *Lactobacillus* status. Alpha diversity of vaginal microbiome decreased markedly in PCOS patients when it was dominated by *Lactobacillus* spp. *Actinomyces* could be a potential biomarker to identify PCOS, but its prediction effect needs to be verified with a larger sample size. *Streptococcus* may have an impact on the pathological changes in PCOS by affecting the female reproductive endocrine environment.

## Data Availability Statement

The datasets presented in this study can be found in online repositories. The names of the repository/repositories and accession number(s) can be found below: NCBI PRJNA775858.

## Ethics Statement

The studies involving human participants were reviewed and approved by Reproductive Medicine Ethics Committee of the Second Affiliated Hospital of Nanjing Medical University. The patients/participants provided their written informed consent to participate in this study.

## Author Contributions

CL, HW, and YQ contributed to conception and design of the study. JY, XZ, and YC supported the clinical epidemiological information collection and analysis. CL performed the statistical analysis and wrote the manuscript. RF and YQ revised and polished the manuscript. All authors contributed to manuscript revision, read, and approved the submitted version.

## Funding

This study was financially supported by National Nature Science Foundation of China (No.81971451, 31900605) and Foundation Research of Jiangsu Province (Nature Science Foundation No. BK 20190654).

## Conflict of Interest

The authors declare that the research was conducted in the absence of any commercial or financial relationships that could be construed as a potential conflict of interest.

## Publisher’s Note

All claims expressed in this article are solely those of the authors and do not necessarily represent those of their affiliated organizations, or those of the publisher, the editors and the reviewers. Any product that may be evaluated in this article, or claim that may be made by its manufacturer, is not guaranteed or endorsed by the publisher.

## Abbreviations

FSH, Follicle-stimulating hormone; LH, luteinizing hormone; E2, estradiol; T, testosterone; BMI, Body mass index; AFC, antral follicle count; LDA, linear discriminant analysis; PCA, principal component analysis; PCoA, principal coordinate analysis; PCOS, polycystic ovary syndrome.
